# Metagenome-Assembled Genome Sequences of Five Strains from the *Microtus ochrogaster* (Prairie Vole) Fecal Microbiome

**DOI:** 10.1128/MRA.01310-19

**Published:** 2020-01-09

**Authors:** Meghan Donovan, Michael D. J. Lynch, Calvin S. Mackey, Grayson N. Platt, Brian K. Washburn, Daniel L. Vera, Darryl J. Trickey, Trevor C. Charles, Zuoxin Wang, Kathryn M. Jones

**Affiliations:** a Department of Biological Science, Florida State University, Tallahassee, Florida, USA; b Department of Psychology, Florida State University, Tallahassee, Florida, USA; c Metagenom Bio, Waterloo, Ontario, Canada; d University of Waterloo, Waterloo, Ontario, Canada; e Department of Biological Science Core Facilities, Florida State University, Tallahassee, Florida, USA; f Department of Biological Science, Center for Genomics and Personalized Medicine, Florida State University, Tallahassee, Florida, USA; University of Maryland School of Medicine

## Abstract

The prairie vole (Microtus ochrogaster) is an important model for the study of social monogamy and dual parental care of offspring. Characterization of specific host species-microbe strain interactions is critical for understanding the effects of the microbiota on mood and behavior. The five metagenome-assembled genome sequences reported here represent an important step in defining the prairie vole microbiome.

## ANNOUNCEMENT

Unusually among rodents, prairie voles form strong mating-induced pair bonds and thus serve as an important model for the study of the neurobiological basis of bonding and associated behaviors (1). Although advances have been made in understanding the neurochemical interactions involved in pair bonding (1), the study of the molecular basis of neuroanatomical responses requires the continued use of a model system. The importance of gut microbes for modulating multiple neurochemical interactions along the “microbiota-gut-brain axis” has been established for humans and mice (2, 3). However, there is considerable variation between mammalian hosts in microbe diversity and metabolism that does not necessarily correlate with host phylogeny, even within a clade such as rodents (4, 5). To date, there have been few studies on the prairie vole microbiome (6, 7). Thus, to facilitate studies of the microbial endocrinology (8) of prairie voles, we have determined the full shotgun metagenome of stool samples from 6 voles and produced an unbinned metagenomic coassembly and 5 metagenome-assembled genomes (MAGs).

Stools were collected from 4 female and 2 male voles (age 3 to 9 months) by temporary isolation of each animal in a bedding-free, sanitized cage. Voles were sexually naive and housed in male/male or female/female cage pairs. All experimental procedures were approved by the Florida State University (FSU) Institutional Animal Care and Use Committee and were in accordance with the U.S. National Institutes of Health Guide for the Care and Use of Laboratory Animals (NIH publication number 80‐23).

DNA was prepared from stool samples frozen at −80°C using the MoBio/QIAamp PowerFecal DNA kit, according to the manufacturer’s instructions (Qiagen USA). Genomic DNA was sheared using a Covaris E220 focused ultrasonicator. Libraries were prepared using the NEBNext Ultra II DNA library prep kit for Illumina (New England BioLabs, USA), following the manufacturer’s protocol. Whole-genome shotgun sequencing of libraries (average fragment size, 765 bp) was performed on an Illumina HiSeq 2500 instrument in the FSU College of Medicine Translational Science Laboratory using paired-end 250-base sequence reads. The total numbers of reads were 66,059,128 (vole 1), 53,198,216 (vole 2), 44,221,130 (vole 3), 54,740,608 (vole 4), 41,707,738 (vole 5), and 48,354,762 (vole 6). Additional sequencing performed on the HiSeq instrument with paired-end 200-base sequence reads generated 125,776,266 (vole 4) and 173,617,670 (vole 6) reads. Read quality control was performed using standard pnnl-atlas v.1.0.35 (9) filtering. The coassembly of all 8 sequence runs and the binning was managed using SqueezeMeta v.1.1.2 (10). Coassembly was performed on reads merged before assembly using Megahit v.1.1.2 (11) (see Table 1 for coassembly details). Data were binned as contigs after coassembly. Binning was performed with MaxBin v.2.2.6 (12) (producing 235 bins) and with metabat2 v.2.12.1 (13) (producing 38 bins). Bins were subsequently processed using DAS Tool v.1.1.1 (14), producing 77 bins. Five bins with high percent completion and low percent contamination were chosen for immediate refinement into MAGs using Anvi’o v.5.5.0 (15), according to an online tutorial (http://merenlab.org/data/refining-espinoza-mags/) (16). Quality was assessed with CheckM v.1.0.18 (17). Default parameters were used for all software, unless otherwise specified.

**TABLE 1:** Characteristics of MAGs and vole stool coassemblies

Characteristic	Data for:													
	MAG						Stool sample from vole no. (sex):							
	MAG13	MAG21	MAG84	MAG226	MAG234	Coassembled_Vole1-6_2017^*a*^	1 (female)	2 (female)	3 (female)	4 (female)	4 (female) (resequencing)	5 (male)	6 (male)	6 (male) (resequencing)
Taxon	*Bacteroidales*	*Bacteroidales*	*Bacteroidales*	*Clostridia*	*Firmicutes*	NA	Prairie vole	Prairie vole	Prairie vole	Prairie vole	Prairie vole	Prairie vole	Prairie vole	Prairie vole
Genome size or no. of bases (Mb)^*b*^	2.1	2.42	2.67	3.11	2.99	269,886,203^*c*^	16.5	13.3	11.1	13.7	25.2	10.4	12.1	33.2
G+C content (%)	40.89	48.39	49.28	38.34	41.89	ND	47	46.8	47.2	47.7	47.7	46.6	46.6	46.7
No. of contigs	213	147	244	348	318	1,551,628								
*N*_50_ (bp)	15,620	26,297	15,553	17,329	15,004	960								
No. of genes identified	1,934	2,092	2,450	3,216	2,957	ND								
No. of tRNAs	34	36	38	44	43	ND								
Completion (%)^*d*^	90.65	90.65	92.09	89.21	94.96	NA								
Estimated redundancy, single-copy core genes (%)^*d*^	2.16	0.72	2.16	0	0	NA								
Estimated contamination (%)^*e*^	1.26	1.15	5.51	1.68	3.38	NA								
Strain heterogeneity (%)^*e*^	50	50	88	66.67	76.92	NA								
GenBank accession no. for sample	WFMC00000000	WFMB00000000	WFMA00000000	WFLZ00000000	WFLY00000000	WFLX000000000								
Platform							Illumina paired end	Illumina paired end	Illumina paired end	Illumina paired end	Illumina paired end	Illumina paired end	Illumina paired end	Illumina paired end
Library name							v1_NEB6_GCCAAT_May2017	v2_NEB1_ATCACG_May2017	v3_NEB2_CGATGT_May2017	v4_NEB3_TTAGGC_May2017	vole4_NEB3_TS1_TTAGGC_Oct2017_deep	v5_NEB4_TGACCA_May2017	v6_NEB5_ACAGTG_May2017	v6_NEB5_ACAGTG_May2017
SRA accession no. for raw reads							SRR9122728	SRR9129758	SRR9130027	SRR9130028	SRR9130365	SRR9130099	SRR9130108	SRR9335148
SRA accession no. for expt							SRX5896710	SRX5903730	SRX5903999	SRX5904000	SRX5904189	SRX5904051	SRX5904060	SRX6101484

a ND, no data; NA, not applicable.

b Genome size is provided for MAGs, and the number of bases is provided for vole stool coassemblies.

c Total number of merged reads.

d From Anvi’o v.5.5.0 (15).

e From CheckM v.1.0.18 (17).

Anvi’o estimated all but one of the MAGs at >90% completeness (see Table 1). Recovery of rRNA genes was poor, which is not unusual for MAGs due to the difficulty of assembling these sequences (18). However, tRNAscan-SE (19) detected 34 to 44 tRNA genes in all of the MAGs, with predicted anticodons for 16 to 20 amino acids. These data will be extremely useful in studies of metabolic functions in the vole microbiome and for comparison with other rodent models.

### Data availability.

The MAG sequences and associated experiment and run data have been deposited in GenBank under BioProject accession number PRJNA449069 and the GenBank and SRA accession numbers given in Table 1.
